# Social differences in COVID-19 vaccination status – Results of the GEDA 2021 study

**DOI:** 10.25646/11268

**Published:** 2023-04-25

**Authors:** Susanne Bartig, Stephan Müters, Jens Hoebel, Nora Katharina Schmid-Küpke, Jennifer Allen, Claudia Hövener

**Affiliations:** 1 Robert Koch Institute, Berlin Department of Epidemiology and Health Monitoring; 2 Robert Koch Institute, Berlin Department of Infectious Disease Epidemiology

**Keywords:** COVID-19, VACCINATION, SOCIAL DETERMINANTS, HEALTH INEQUALITY, GEDA 2021

## Abstract

**Background:**

The COVID-19 vaccination is a key measure to contain the pandemic. It aims to restrict new infections and to reduce severe courses of the disease. This paper examines the influence of various social determinants on COVID-19 vaccination status.

**Methods:**

The analyses are based on data from the study German Health Update (GEDA 2021), a nationwide telephone-based survey of the adult population in Germany, which was conducted between July and December 2021. In addition to bivariate analyses, the association between the COVID-19 vaccination status and the social determinants was examined using Poisson regression.

**Results:**

A total of 86.7% of people aged 18 years and older who participated in GEDA 2021 have been received at least one dose of COVID-19 vaccine. Social differences are evident: The proportion of people vaccinated against COVID-19 increases with age, income and higher education group. Lower vaccination rates are found among people with a history of migration, people living in rural areas and people from East Germany. An age-differentiated analysis shows that the social differences in COVID-19 vaccination uptake are lower among those aged 60 years and older.

**Conclusions:**

The presented results should be considered when designing targeted interventions to overcome potential barriers to COVID-19 vaccination uptake. Further research is needed regarding the explanatory factors for the social differences in vaccination behaviour, such as structural and group-specific barriers or psychological determinants.

## 1. Introduction

The SARS-CoV-2 virus (Severe Acute Respiratory Syndrome Coronavirus 2) was first detected in December 2019 in Wuhan, China. In early 2020, SARS-CoV-2, which causes the disease COVID-19, spread rapidly around the world [1–3]. In addition to nonpharmaceutical interventions, such as general contact restrictions, keeping a minimum distance or wearing a mouth-nose covering [4, 5], vaccination against COVID-19 is an essential measure for containment of the pandemic. The vaccination aims to reduce severe courses of the disease and deaths and to prevent the transmission of SARS-CoV-2 throughout the population [5, 6].

Following the COVID-19 vaccination campaign began in late December 2020 with BioNTechPfizer’s Comirnaty^®^ vaccine [7], five additional vaccines were successively approved in Germany by October 2022 [8, 9]. However, because the vaccines’ availability was initially limited, some groups were prioritized at the beginning of the vaccination campaign: In addition to people with an increased risk of severe courses of the disease, priority was primarily given to people with a high occupational risk of exposure to SARS-CoV-2 or who were in close contact with people at risk [5, 6]. This prioritization recommendation was lifted in June 2021 [10]. Since then, everyone aged 18 years and older has had the opportunity to be vaccinated.

However, various studies in Germany indicate social differences in willingness to be vaccinated and in COVID-19 vaccination uptake. For example, the willingness to be vaccinated against COVID-19 is more pronounced among older people, people with higher level of education and higher socioeconomic status, and people without a migration background [11, 12]. Analyses from the COMPASS survey (Corona-Online-Meinungs-Panel-Survey-Spezial) of the German Institute for Economic Research (DIW) for the survey month of July 2021 also show that COVID-19 vaccination rates are associated with sociodemographic and socioeconomic determinants: Vaccination rates increase significantly with rising age, educational level and household income [13].

The COVID-19 vaccination rate monitoring in Germany (COVIMO), which has been conducted regularly by the Robert Koch Institute (RKI) since early 2021, collects information on the willingness to get vaccinated and vaccine acceptance of the German-speaking population aged 18 years and older through a nationwide telephone-based survey. According to the results of the eighth survey (data collection: 15.09.–18.10.2021), COVID-19 vaccination rates are lower among people seeking for work or working short-time jobs and those living in the southern federal states of Germany [14]. The ninth survey (data collection: 04.11.–18.12.2021) focused on Germany as an ‘immigration society’ and the interviews previously conducted in German were supplemented by five other languages. The results show that people without a history of migration have a higher vaccination rate, but willingness to get vaccinated of the unvaccinated is higher among those with a history of migration [15]. The COVID-19 Snapshot Monitoring (COSMO), which has been conducted as online survey weekly or monthly since March 2020, and the ‘Begleitforschung zur Kommunikation der Corona-Schutzimpfung in Deutschland (CoSiD)’ by the Federal Centre for Health Education (BZgA) also indicate differences in the COVID-19 vaccination uptake depending on sociodemographic and socioeconomic factors [16, 17].

This paper aims to examine the influence of different social determinants on the COVID-19 vaccination status based on the sixth wave of the German Health Update (GEDA 2021). GEDA 2021 allows a differentiated description of COVID-19 vaccination rates according to a variety of sociodemographic, socioeconomic and regional characteristics after lifting the vaccination prioritization of particularly vulnerable groups. In addition to descriptive analyses, this paper identifies – by means of multivariate analyses – relevant social determinants of the COVID-19 vaccination status. In comparison to earlier studies, an age-differentiated presentation of the vaccination rates for the selected social determinants is also provided.

**GEDA 2021**

Sixth follow-up survey of the German Health Update

**Data holder:** Robert Koch Institute

**Objectives:** Provision of reliable information on the health status, health behaviour and health care of the population living in Germany and their changes in the course of the SARS-CoV-2 pandemic.

**Study design:** Cross-sectional telephone survey

**Population:** German-speaking population aged 16 years and older living in private households that can be reached via landline or mobile phone

**Sampling:** Random sample of landline and mobile telephone numbers (dual-frame method) from the ADM sampling system (Arbeitskreis Deutscher Markt- und Sozialforschungsinstitute e.V.)

**Sample size:** 5,030 respondents

**Study period:** July 2021 to December 2021

**GEDA survey waves:**

► GEDA 2009
► GEDA 2010
► GEDA 2012
► GEDA 2014/2015-EHIS
► GEDA 2019/2020-EHIS
► GEDA 2021

Further information in German is available at www.geda-studie.de

## 2. Methods

### 2.1 Study design and sample

‘German Health Update (GEDA)’ is a nationwide cross-sectional survey of the resident population in Germany, which has been conducted regularly since 2008 by the Robert Koch Institute (RKI) on behalf of the Federal Ministry of Health and is part of the health monitoring at the RKI [18–20]. In addition to the health status, GEDA collects information on health behaviour, the living conditions of the population and the utilisation of health care services.

The sixth follow-up survey (GEDA 2021) was conducted between July and December 2021 as a telephone interview using a programmed, fully structured questionnaire (i.e. Computer Assisted Telephone Interview, CATI). The follow-up of the GEDA study as a continuous survey enabled the establishment of a flexible surveillance instrument at the RKI. In addition to so-called core modules, current pandemic-related topics such as vaccination behaviour, previous SARS-CoV-2 infections, domestic quarantine as well as special risk factors for SARS-CoV-2 infection could be flexibly integrated into the survey. The data allow analyses of changes in health status (including mental health), health behaviour and the utilisation of health care services over the course of the pandemic [21].

The survey is based on a random sample of landline and mobile telephone numbers (dual-frame method) [22]. According to the Framework Regulation for European Statistics agreed at EU level, the study population comprised the German-speaking population aged 16 years and older living in private households whose usual place of residence at the time of data collection was in Germany. This definition does not include collective households such as hospitals, nursing or residential homes, boarding houses, etc. [23]. The telephone sampling system of the ADM (Arbeitskreis Deutscher Markt- und Sozialforschungsinstitute e.V.) was used for sampling procedure, which allows for (almost) complete coverage of the study population [24]. The data was collected by interviewers from an external market and social research institute (USUMA GmbH). Staff from the RKI monitored the entire survey process through continuous supervision and in the form of comprehensive field monitoring. The aim of the study was to interview at least 1,000 people per wave in a monthly wave design. Each wave can be analysed separately.

A total of 5,030 people (2,608 women, 2,422 men) participated in GEDA 2021 providing completed interviews. The combined Response Rate 3 (RR3) was calculated according to the standards of the American Association for Public Opinion Research (AAPOR) [25] and range between 17.6% (wave 1) and 22.5% (wave 4). The RR3 reflects the proportion of realised interviews relative to all likely households in the study population.

### 2.2 Indicators

The COVID-19 vaccination status was captured in GEDA 2021 by asking: ‘Have you already been vaccinated against the coronavirus disease COVID-19?’ (Response categories: ‘Yes’, 'No'). Associations between the uptake of COVID-19 vaccination (at least once) and social determinants were examined using the following indicators:

#### Gender and age

Gender at birth was used to describe gender differences, i.e. the gender stated on the birth certificate (according to self-reports). The age of the respondents was categorised into the following groups: 18 to 39 years, 40 to 59 years and 60 years or older.

#### Education

To measure the educational status, study participants’ educational and vocational qualifications were categorised into low (ISCED 0–2), medium (ISCED 3–4) and high (ISCED 5–8) education groups according to the 2011 version of the International Standard Classification of Education (ISCED 2011) [26].

#### Income

Based on the self-reported monthly net income of the study participants’ households, the net equivalent income was calculated using the new equivalence scale of the Organisation for Economic Co-operation and Development (OECD), taking into account the composition of the household in terms of size and age structure [27], to consider savings from sharing expenses in multi-person households. Missing income information was imputed using regression analysis procedures with information on age, gender, composition of the household, education, employment status, occupational position as well as regional information on unemployment and income tax. In the following, low- (quintile 1), medium- (quintiles 2–4) and high-income groups (quintile 5) were generated for the analyses.

#### Current region of residence

As an indicator of the current region of residence, the study participants’ information on the federal state in which they currently live was used. For the analyses, the respective federal states in East and West Germany were combined, with the exception of Berlin as a separate category.

#### Urban versus rural

In order to examine whether living in an urban or rural area has an influence on the COVID-19 vaccination uptake, the four settlement-structural district types of the Federal Institute for Research on Building, Urban Affairs and Spatial Development (BBSR) [28] were assigned to the categories urban (large cities without districts, urban districts) and rural (rural districts with beginning densification, sparsely populated rural districts). The districts were self-reported by the respondents.

#### History of migration

In addition to socioeconomic and regional characteristics, the COVID-19 vaccination status is presented by the (non-)presence of a history of migration. This is operationalized on the basis of the country of birth of the respondent and his/her parents as ‘without history of migration’, ‘own history of migration’ (people who have immigrated themselves) and ‘parental history of migration’ (at least one parent was not born in Germany). The term ‘history of migration’ as used in this paper and recommendations for analysing migration-related determinants in public health research are described in detail elsewhere [29].

### 2.3 Statistical analyses

In the present article, the proportions of people who had received at least one COVID-19 vaccination are reported by selected social determinants and with 95% confidence intervals (95% CI). A significant difference between groups (determined by chi-square tests) is assumed if the p-value is less than 0.05. In the following, only the results of the descriptive analyses that are statistically significant according to respective chi-square test are reported here. To complement the bivariate analyses, multivariate adjusted prevalence ratios (PR) with corresponding 95% CIs and p-values were calculated using Poisson regression to identify relevant associations between the uptake of COVID-19 vaccination and social determinants. All social determinants were included in the regression analysis and adjusted for the survey month. The results of the Poisson regression are presented as a Forest plot. Statistically significant associations with the vaccination status are assumed if the respective 95% CIs of the social determinants do not include a value of 1.

The analyses were performed with a weighting factor to correct for deviations of the sample from the population structure. In addition to the design weighting for different selection probabilities (mobile and landline), the sample was adjusted to the official population figures of the Federal Statistical Office with regard to age, gender, federal state, district type (as of: 31.12.2020) and education (micro census 2018). Missing values in the investigated variables were excluded from the bivariate and multivariate analyses.

All analyses were conducted with StataSE 17.0 (Stata Corp., College Station, TX, USA, 2015) using the survey procedures.

## 3. Results

###  

#### Sample description

The analyses are based on data from 4,954 respondents aged 18 years and older (2,576 women, 2,378 men) providing valid information on their COVID-19 vaccination status. Only participants aged 18 years and older were included in the analyses, as the recommendation for COVID-19 vaccination of those between 12 and 17 years of age was only given during the survey period in August 2021 [30].

Table 1 shows the distribution of the sample by the selected social determinants. Of the 4,954 participants included in the analyses, about half are women (50.9%). The median age among the study population is 52 years (range: 18–97 years). People who belong to the low (18.1%) and high (25.5%) education groups are less likely to be represented in the sample than those of the medium education group (56.4%). The median of the monthly equivalized disposable income is around 2,028 Euro. The vast majority of the participants reside in West Germany (80.4%) and almost two thirds of the respondents live in urban areas (68.6%). Three out of four participants (73.0%) do not have a history of migration; people with an own (12.1%) or parental (14.9%) history of migration are almost equally represented in the sample (Table 1).

#### Results of the bivariate analyses

**Figure 1:**
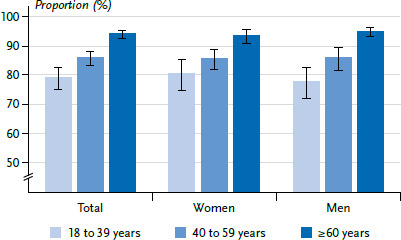
Proportion of people vaccinated against COVID-19 (at least once) by age group and gender (n=4,954) Source: GEDA 2021

Overall, 86.7% of participants aged 18 years and older report having been vaccinated against COVID-19 at least once (women 87.3%, men 86.2%). The proportion of those vaccinated against COVID-19 increases with age (Figure 1, Table 2): While 79.1% of the youngest age group (18 to 39 years) are vaccinated, the proportion is highest (94.2%) among respondents aged 60 years and older, which were initially prioritized in the vaccination campaign. There are no evident differences by gender (Figure 1).

The results show a socioeconomic gradient in COVID-19 vaccination status (Table 2). People in the lower education group are less likely to be vaccinated against COVID-19 (82.5%) than those from the medium (86.0%) and high (91.5%) education groups. A similar socioeconomic patterning of COVID-19 vaccination is observed for income level: Whereas 78.1% of participants belonging to the low income group are vaccinated against COVID-19, the proportion is 87.5% for the medium- and 93.0% for the high-income group.

Moreover, there is a detectable East vs. West gradient in the COVID-19 vaccination status. The proportion of people vaccinated against COVID-19 in West Germany (88.0%) is almost 10 percentage points higher compared to those from East Germany (79.8%). In Berlin, the vaccination rate of 87.1% is at a similar level as in West Germany. The East-West difference in COVID-19 vaccination status is more pronounced among male participants (11.7 percentage points) than among women (4.8 percentage points) (Annex Table 1). Furthermore, people living in rural areas (83.5%) are less likely to be vaccinated against COVID-19 than those living in urban settings (88.6%). COVID-19 vaccination status also varies with regard to the (non-)presence of a history of migration. In particular, people who immigrated themselves (79.1%) have a lower COVID-19 vaccination rate than those without a history of migration (89.0%).

**Figure 2:**
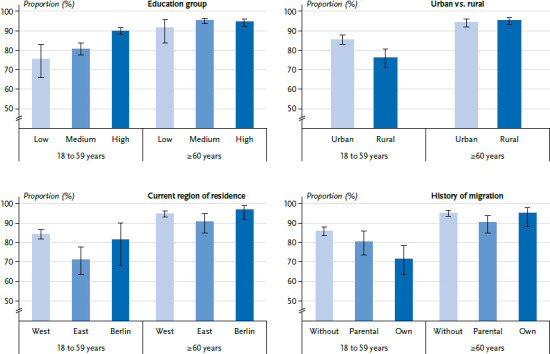
Proportion of people vaccinated against COVID-19 (at least once) by selected social determinants and age group Source: GEDA 2021

Differentiating by age, the social differences in COVID-19 vaccination status are less pronounced among those aged 60 years and older than among 18 to 59-year-olds (Figure 2). Regarding the educational level, it can be observed that COVID-19 vaccination rates in the younger age group (18 to 59 years) increase with higher level of education. In comparison, the proportion of COVID-19 vaccinated people in the older age group (60 years and older) is at a similar level in the different education groups. Another example of lower social differences in COVID-19 vaccination status in the age group of 60 years and older is shown for the comparison of East versus West Germany: While there are nearly no differences in the vaccination rate between respondents in East and West Germany in the older age group (West: 94.9%, East: 91.0%), the difference among 18 to 59-year-olds is 13 percentage points (West: 84.4%, East: 71.4%).

#### Results of multivariate analysis

**Figure 3:**
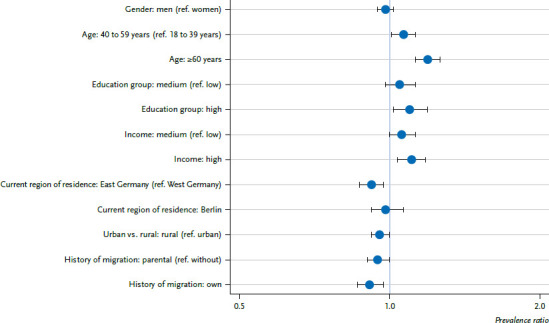
Determinants of COVID-19 vaccination (at least once), results of Poisson regression analysis (n=4,671) Source: GEDA 2021

The multivariate Poisson regression analysis confirms that an age of 40 years and older is positively associated with the uptake of COVID-19 vaccination (Figure 3, Annex Table 2). In addition, a higher level of education and income are both independently, i.e. under reciprocal statistical control, associated with increasing COVID-19 vaccination uptake.

Considering regional differences, while keeping the investigated social determinants statistically constant, there is also an association with the COVID-19 vaccination status: Respondents from East Germany and people living in rural areas have a lower vaccination rate. Furthermore, having a (own or parental) history of migration remains independently associated to a lower uptake of COVID-19 vaccination according to multivariate control. However, the correlation between vaccination status and living in rural areas (p=0.034) or having a parental history of migration (p=0.039) is less pronounced compared to the other social determinants (Annex Table 2).

## 4. Discussion

This paper examines the influence of selected social determinants on COVID-19 vaccination status in Germany. Based on analyses of the GEDA 2021 study, the results suggest social differences in COVID-19 vaccination. In line with previous studies in Germany, the proportion of COVID-19 vaccinated people increases with age, higher level of education and income [13, 16, 17]. Various international studies also indicate a social gradient in the willingness to be vaccinated and uptake of COVID-19 vaccination [31–39]. In addition, the determinants history of migration, region of residence (East versus West Germany) and living in urban versus rural areas are associated with the uptake of COVID-19 vaccination. Accordingly, people from East Germany, people with an own or parental history of migration and people living in rural areas show a lower COVID-19 vaccination rate. While various surveys in Germany indicate a higher willingness to be vaccinated [11, 40] and vaccination rates for men [13, 41, 42], the present paper – comparable to results of the CoSiD study [17] – finds no gender differences in the COVID-19 vaccination uptake.

Overall, it should be noted that the present paper describes differences by selected sociodemographic, socioeconomic and regional characteristics. However, the results only allow limited conclusions to be drawn about the reasons for the different uptake of COVID-19 vaccination. For example, the presence of a history of migration per se is not the cause of a lower vaccination rate. Rather, it is necessary to consider the underlying mechanisms and explanatory factors that are linked to the respective social determinants and influence the access to and uptake of vaccination. For example, structural (e.g. difficult access to vaccination services) and group-specific barriers (e.g. poor German language skills) or the influence of psychological factors on vaccination behaviour should be taken into account.

People with a history of migration face specific barriers to the uptake of COVID-19 vaccination. The COVID-19 vaccination rate monitoring in Germany as an immigrant society (COVIMO focus survey) showed that German language skills (German as mother tongue or self-assessment of German language skills) explain much of the differences in the COVID-19 vaccination rate between people with and without a history of migration. Moreover, experiences of discrimination in the health or care sector also contribute to explaining the vaccination rate differences [15]. Another result of the focus survey relates to the knowledge about COVID-19 vaccination: Uncertainties regarding the surveyed knowledge items are significantly more common among people with a history of migration than among people without a history of migration [15]. This may be due to the fact that health-related information and services provided by the health system are often not oriented towards the linguistic diversity in Germany [43].

Elderly people have a specific vaccination indication [44] because of their higher risk of severe COVID-19 [6, 45], which can partly explain both the association between age and vaccination rate shown here and the less pronounced social differences in the older age group as compared to the younger age group. In addition, people who are older and those with a higher level of education are more likely to have positive attitudes towards vaccination in general [46], which strengthen the willingness to get vaccinated against COVID-19 [47, 48]. Various studies in Germany also indicate that age and education status are associated with subjectively perceived levels of being informed about vaccination: People of more advanced age and those of higher level of education more often feel (very) well informed about the vaccination [17, 42]. Moreover, higher educational levels are positively associated with health literacy in general [49, 50], i.e. the ability to find, evaluate and use health-related information as the basis for decision-making [51]. Analyses of the data of the third survey of the CoSiD study (data collection: 15.11.–08.12.2021) show that respondents of younger age, with lower educational level and a migration background report lesser health literacy regarding the COVID-19 vaccination [52]. However, international studies suggest that the willingness to be vaccinated is associated with health literacy [53]. According to the higher level of health literacy associated with higher education groups may have a positive influence on the COVID-19 vaccination behaviour.

Against the background of the generally higher vaccination rates in East Germany, e.g. with respect to vaccination against measles or influenza [46, 54–56], the lower COVID-19 vaccination rate compared to West Germany is initially surprising. People who grew up in the GDR are more likely to report positive attitudes towards vaccinations [46]. During the COVID-19 pandemic, more critical attitudes towards the containment measures were found in East German regions, and these are associated, on the one hand, to political attitudes and, on the other hand, to the COVID-19 vaccination rate [57]. Indications of a trend towards a more critical assessment of corona-related measures by parts of the East German population are also evident in the representative survey ‘Einstellungen, Wissen und Verhalten von Erwachsenen und Eltern gegenüber Impfungen (Attitudes, knowledge and behaviour of adults and parents towards vaccinations)’ of the BZgA (data collection: 26.07.–07.09.2021). In addition to showing a lower level of confidence in the safety of COVID-19 vaccination, respondents from East Germany are less likely to agree with the statement that vaccination can contribute to containing the spread of the virus [42].

Psychological factors further contribute to explaining (social) differences in COVID-19 vaccination uptake. The established ‘5C’ model based on five ‘psychological antecedents of vaccination’: confidence (e.g. in the safety and effectiveness of the vaccine), complacency (perceived risk of the disease), constraints (structural and psychological barriers), calculation (risk-benefit analysis) and collective responsibility (willingness to protect others) [58, 59]. Confidence in the safety and effectiveness of vaccination is the most stable predictor of vaccination behaviour across all COVIMO surveys. Results of the first wave of the COSMO Panel Study (December 2021) also show that non-vaccinated respondents not only have less confidence in the safety of the vaccination, but also lack confidence in institutions (Robert Koch Institute, science) or decision-makers such as the government. But the major barrier to getting vaccinated is the fear of side effects [16]. International research suggests similar results for the impact of psychological factors on the COVID-19 vaccination behaviour: In addition to differences in risk perception, variations in vaccination are mainly explained by concerns about the safety and effectiveness of vaccination [47, 48, 60–63]. Furthermore, international studies show that misinformation resulting from social media has a negative impact on confidence in the safety of the vaccination and is the cause of the reduced willingness to get vaccinated against COVID-19 [37, 53, 64, 65].

The influence of psychological factors on vaccination behaviour also differs by social determinants: Accordingly, the results of the representative survey of the BZgA (data collection: 26.07.–07.09.2021) show that older people (60 years and older) have a greater sense of responsibility for the community and confidence in the safety of vaccination, and carefully weigh up benefits and risk of vaccination. The COSMO study also indicates that age and the psychological factors confidence and cost-benefit calculation are associated [41]. With regard to the (non-)presence of a history of migration, differences are evident in the influence of collective responsibility on vaccination behaviour. While there is no effect for people without a history of migration, people with a history of migration are more likely to be vaccinated the more they view vaccination as a community measure to prevent the spread of COVID-19 [15].

###  

#### Strengths and limitations

A major strength of the study is the ability to differentiated analyses of COVID-19 vaccination uptake according to a large variety of social determinants. In addition to age, gender, income and education, other relevant factors of vaccination behaviour were considered in the analyses for this paper, such as history of migration and selected regional characteristics (East versus West Germany, urban versus rural). Simultaneous integration of the influencing factors in the Poisson regression analysis allowed the reciprocal control of the effects on the COVID-19 vaccination status. Compared to vaccination rates based on reported data, surveys such as GEDA 2021 can be used to identify the vaccination potential in different population groups and, by including further information, to design targeted measures aiming to overcome possible vaccination barriers. Accordingly, surveys in which the vaccination rates of different population groups are collected are an important supplement to reported data.

Nevertheless, several limitations should be noted. Surveys aimed at directly capturing COVID-19 vaccination behaviour are assumed to have a biased sample. For example, people with positive attitudes towards vaccination are more likely to participate in such surveys than people who are less willing to get vaccinated and are therefore under-represented in the sample [16, 66]. Similar to other surveys that measure the COVID-19 vaccination status, the vaccination rate must be assumed to be overestimated in GEDA 2021 due to social desirability in the response behaviour. An experimental study shows that the COVID-19 vaccination rate is overestimated as a result of social desirability when the vaccination status is collected directly [67]. In contrast, the COVID-19 Digital Vaccination Coverage Monitoring (DIM), the surveillance system for COVID-19 vaccinations [68], is assumed to underestimate the vaccination rates by a few percentage points. Given the under-reporting of vaccinating agencies, the COVID-19 vaccinations reported in the DIM are to be regarded as minimum vaccination rates [66, 69]. Deviations between vaccination rates collected in surveys and vaccination rates calculated via the surveillance system are therefore to be expected, but the impact of social desirability on the response behaviour would be difficult to determine. Other possible explanations for the discrepancy between the vaccination rates from the DIM and the COVIMO telephone-based survey of the German-speaking adult population regarding the willingness and acceptance of vaccination, which has a comparable study design as GEDA 2021, have already been discussed in the seventh COVIMO Report [66].

Another limitation is that the case numbers for differentiated analyses by individual subgroups, for example 18 to 59-year-olds who belonging to the low education group or 18 to 59-year-olds having an own history of migration, are rather small. Consequently, comparisons between these subgroups have limited interpretability.

A further limitation of the study is that the survey was conducted only in German and therefore people without German language skills were excluded from the survey. As a result, this article describes the proportion of vaccinated people in the German-speaking population aged 18 years and older. Against this background, the vaccination rate differences between people with and without a history of migration shown here may be underestimated. Furthermore, possible explanations for the differences in COVID-19 vaccination status cannot be comprehensively investigated on the basis of the study.

Telephone-based surveys also show that people belonging to lower education groups are less likely to participate in the study than those of higher education groups. This can increase the risk of biased results caused by the systematic non-participation of various population groups (non-response bias). Using appropriate weighting procedures (see 2.3 Statistical analyses) this is countered (calibration/adjustment weighting) [20].

#### Conclusion and outlook

Based on analyses of the GEDA 2021 study, it was shown that the COVID-19 vaccination status differs by the selected social determinants. In order to design target group-specific interventions to overcome potential barriers to vaccination, further research is needed considering possible mechanisms and explanatory factors for the different uptake of a COVID-19 vaccination in the various population groups. Thus, it is not the educational status, the current region of residence or a history of migration per se that is decisive for the COVID-19 vaccination status, but other factors influencing vaccination behaviour must be taken into account, such as group-specific and structural barriers or psychological determinants, such as confidence in the safety of the vaccination.

The seroepidemiological ‘Corona Monitoring Nationwide (RKI-SOEP-2)’ study, which was conducted from November 2021 to February 2022 as a cooperative project involving the RKI, the Socio-Economic Panel (SOEP) at the German Institute for Economic Research (DIW), the Institute for Employment Research (IAB) and the Research Centre of the Federal Office for Migration and Refugees (BAMF-FZ) allows various factors influencing COVID-19 vaccination behaviour to be taken into account [70, 71]. A main goal was to estimate the proportion of the German population that had been vaccinated against COVID-19 and/or had been infected with SARS-CoV-2 at the turn of the year 2021/2022. The study consisted of self-collected dry blood samples to detect antibodies against SARS-CoV-2 virus and a detailed self-administered questionnaire of the vaccination and infection status. Detailed analyses are currently being conducted, including vaccination and infection status differentiating by the (non-)presence of a history of migration as well as socioeconomic status (education, income) and explanatory factors for the vaccination rate differences in various population groups.

The limitation that GEDA 2021 was only conducted in German is addressed by the multilingual survey ‘German Health Update: Fokus’ (GEDA Fokus), which was conducted from November 2021 to May 2022 among people with Croatian, Italian, Polish, Syrian and Turkish citizenship at the RKI [72]. The study aimed to collect comprehensive information on the health status, health behaviour, living conditions and the utilisation of health care services. Questions on SARS-CoV-2 infection and COVID-19 vaccination status were also a thematic focus of the survey. Results of the association between the COVID-19 vaccination uptake and sociodemographic, health- and migration-related factors were published in early 2023 [73].

Against the background of the socially unequal distribution of SARS-CoV-2 infection risk and of the severe courses of COVID-19 [44, 74, 75], the social inequality in COVID-19 vaccination status indicated in the present article highlights the need for health policies. To ensure equal access to vaccination offers for all people and to counteract the increased risk of infection and mortality of socially disadvantaged groups, low-threshold, lifeworld-related and targeted infection protection and vaccination offers, which are adapted to the living conditions of the people and are accompanied by proactive offers (outreach), are required. This includes the involvement of (multilingual) mediators or key people from the communities to disseminate information about the offers and to increase the acceptance of vaccinations [75]. The fact that community-oriented interventions have a positive impact on the vaccination campaign has been demonstrated in Bremen [76], Bad Nauheim [77, 78] or Berlin [79].

## Key statement

- Increasing age is associated with higher uptake of COVID-19 vaccination.
- The proportion of people vaccinated against COVID-19 increases with higher levels of education and income.
- People from East Germany are less likely to be vaccinated against COVID-19.
- Social differences in COVID-19 vaccination status are lower among those aged 60 years and older than in 18 to 59-year-olds.
- There is a need to research the underlying mechanisms and explanatory factors of social differences in vaccination behaviour, such as specific access barriers or psychological determinants.

## Figures and Tables

**Table 1:** Sample description by selected social determinants (n=4,954) Source: GEDA 2021

Number of cases (n)		Weighted sample (%)
**Gender**		
Women	2,576	50.9
Men	2,378	49.1
**Age group**		
18–39 years	901	31.6
40–59 years	1,662	32.6
≥60 years	2,391	35.8
**Education group**		
Low	240	18.1
Medium	2,081	56.4
High	2,613	25.5
Missing	20	-
**Income**		
Low	609	19.9
Medium	2,921	60.6
High	1,424	19.5
**Current region of residence**		
West Germany	3,799	80.4
East Germany	827	15.1
Berlin	325	4.4
Missing	3	-
**Urban versus rural**		
Urban	3,386	68.6
Rural	1,354	31.4
Missing	214	-
**History of migration**		
Without	3,650	73.0
Parental	713	14.9
Own	524	12.1
Missing	67	-

**Table 2:** Proportion of people vaccinated against COVID-19 (at least once) by social determinants Source: GEDA 2021

%		(95% CI)
**Gender**		
Women	87.3	(84.8–89.3)
Men	86.2	(83.6–88.4)
Age group^*^		
18–39 years	79.1	(74.9–82.8)
40–59 years	85.8	(82.9–88.3)
≥60 years	94.2	(92.4–95.6)
Education group^*^		
Low	82.5	(76.2–87.4)
Medium	86.0	(83.6–88.1)
High	91.5	(89.9–92.9)
Income^*^		
Low	78.1	(72.5–82.8)
Medium	87.5	(85.4–89.4)
High	93.0	(90.7–94.8)
Current region of residence^*^		
West Germany	88.0	(86.1–89.7)
East Germany	79.8	(74.7–84.1)
Berlin	87.1	(77.4–93.0)
Urban versus rural^*^		
Urban	88.6	(86.6–90.4)
Rural	83.5	(79.9–86.6)
History of migration^*^		
Without	89.0	(87.1–90.7)
Parental	83.3	(78.1–87.5)
Own	79.1	(72.7–84.3)

CI = confidence interval,

^*^ significant difference according to Chi-square test

## Appendix

**Annex Table 1:** Proportion of people vaccinated against COVID-19 (at least once) by gender and social determinants Source: GEDA 2021

	Women		Men	
	%	(95% CI)	%	(95% CI)
**Education group**				
Low	83.5	(75.1–89.4)	81.0	(70.2–88.6)
Medium	87.7	(84.6–90.2)	84.2	(80.4–87.4)
High	90.6	(87.8–92.8)	92.2	(90.1–93.9)
**Income**				
Low	78.1	(70.1–84.4)	78.1	(69.8–84.5)
Medium	88.6	(85.8–90.9)	86.4	(83.0–89.2)
High	93.1	(89.0–95.7)	92.9	(90.0–95.0)
**Current region of residence**				
West Germany	88.1	(85.4–90.3)	87.9	(85.0–90.3)
East Germany	83.3	(76.4–88.5)	76.2	(68.2–82.7)
Berlin	85.1	(66.8–94.2)	89.3	(80.7–94.3)
**Urban versus rural**				
Urban	88.7	(85.8–91.1)	88.4	(85.4–90.9)
Rural	84.8	(79.8–88.7)	82.2	(76.6–86.7)
**History of migration**				
Without	89.7	(87.0–91.9)	88.3	(85.4–90.7)
Parental	84.8	(77.9–89.8)	81.6	(73.1–87.9)
Own	78.9	(69.6–86.0)	79.3	(69.9–86.3)

CI = confidence interval

**Annex Table 2:** Determinants of COVID-19 vaccination (at least once), results of the Poisson regression analysis (n=4,671) Source: GEDA 2021

PR (95% CI)		p-value
**Gender**		
Women	Ref.	
Men	0.98 (0.94–1.02)	0,270
**Age group**		
18–39 years	Ref.	
40–59 years	1.07 (1.01–1.13)	**0,027**
≥60 years	1.19 (1.13–1.25)	**<0,001**
**Education group**		
Low	Ref.	
Medium	1.05 (0.98–1.13)	0,198
High	1.10 (1.02–1.19)	**0,009**
**Income**		
Low	Ref.	
Medium	1.06 (1.00–1.13)	0,050
High	1.11 (1.04–1.18)	**0,001**
**Current region of residence**		
West Germany	Ref.	
East Germany	0.91 (0.85–0.97)	**0,004**
Berlin	0.98 (0.91–1.07)	0,661
**Urban versus rural**		
Urban	Ref.	
Rural	0.95 (0.91–1.00)	**0,034**
**Migration history**		
Without	Ref.	
Parental	0.94 (0.89–1.00)	**0,039**
Own	0.90 (0.84–0.97)	**0,004**

PR = Prevalence ratio, Ref. = Reference group, p-values from multivariate Poisson regression analyses with two-way control of the social determinants and adjusted for survey month **bold** = statistically significant as compared to the reference group
